# “The times they are a-changin’”: A longitudinal, mixed methods case study of a hospital transformation

**DOI:** 10.1371/journal.pone.0272251

**Published:** 2022-10-25

**Authors:** Chiara Pomare, Brett Gardiner, Louise A. Ellis, Janet C. Long, Kate Churruca, Jeffrey Braithwaite

**Affiliations:** Centre for Healthcare Resilience and Implementation Science, Australian Institute of Health Innovation, Macquarie University, North Ryde, NSW, Australia; Brown University, UNITED STATES

## Abstract

**Background:**

Changes to hospital infrastructure are inevitable in ever-evolving healthcare systems. The redevelopment of hospitals and opening of new buildings can be a complex and challenging time for staff as they must find ways to deliver safe and high-quality care while navigating the complexities and uncertainties of change. This study explores the perspectives and experiences of staff and patients before and after the opening of a new hospital building as part of a large public hospital redevelopment in Sydney, Australia.

**Methods:**

The study comprised a longitudinal mixed methods case study design. Methods included two rounds of staff surveys (n = 292 participants), two rounds of staff interviews (n = 66), six rounds of patient surveys (n = 255), and analysis of hospital data at tri-monthly intervals over two years. Data were compared before (2019) and after (2020) a new hospital building opened at a publicly funded hospital in Sydney, Australia.

**Results:**

Four key themes and perspectives emerged from the interviews including change uncertainty, communication effectiveness, staffing adequacy and staff resilience. Significant differences in staff perceptions of change readiness over time was identified. Specifically, perceptions that the organisational change was appropriate significantly decreased (2019: 15.93 ± 3.86; 2020: 14.13 ± 3.62; *p* < .001) and perceptions that staff could deal with the change significantly increased (2019: 17.30 ± 4.77; 2020: 19.16 ± 4.36; *p* = .001) after the building opened compared to before. Global satisfaction scores from patient survey data showed that patient experience significantly declined after the building opened compared to before (2020: 81.70 ± 21.52; 2019: 84.43 ± 18.46)), *t*(254) = -64.55, *p* < 0.05, and improved a few months after opening of the new facilities. This coincided with the improvement in staff perceptions in dealing with the change.

**Conclusions:**

Moving into a new hospital building can be a challenging time for staff and patients. Staff experienced uncertainty and stress, and displayed practices of resilience to deliver patient care during a difficult period of change. Policy makers, hospital managers, staff and patients must work together to minimise disruption to patient care and experience. Key recommendations for future hospital redevelopment projects outline the importance of supporting and informing staff and patients during the opening of a new hospital building.

## Background

“*People don’t resist change*. *They resist being changed”*– Jack Welch

“*Culture does not change because we desire to change it*. *Culture changes when the organization is transformed–the culture reflects the realities of people working together everyday”*– Frances Hesselbein

Healthcare is increasingly recognised as a complex system where change is constant, yet emergent and stochastic [1–3]. Hospitals undergo frequent organisational change including adjustments to workforce and governing structures [4], implementation or withdrawal of medical technologies and equipment [5], and alterations to hospital infrastructure through redevelopment [6, 7].

Hospital redevelopment, involving the re-design and refurbishment of older hospital infrastructure or the addition of new infrastructure, is an example of a recurring and inevitable organisational change in healthcare. That is, as populations and demand for healthcare services grow [8], we need to continually consider how to redevelop, modernise, and alter existing hospital infrastructure to face the many challenges of contemporary healthcare systems; such as the growing and ageing global population, medical advances, outdated and inadequate infrastructure, and concerns about the quality and safety of current health services [9–11]. Indeed in Australia [6, 12, 13], as in many other high-income countries [7], hospital redevelopment has become a booming industry, and is set to continue. In New South Wales alone, there are more than 100 major health capital projects (i.e., projects over AUD$10 million) currently in train [14].

Redeveloping a hospital is a protracted process whereby there are large-scale changes to the physical environment (i.e., services ultimately move to a new hospital environment) while hospital staff must continue to deliver care to patients. Indeed, this period of change has been described by terms such as *unsettling* and *stressful* for hospital staff as they strive to deliver safe and high-quality care across a fluctuating landscape alongside construction-related infrastructural activities [15]. Major changes to a hospital building can also be a challenging time for patients as they must deal with noise and disruption from construction which can negatively influence their experiences while receiving care [16]. On the other hand, large-scale hospital change can also be positive and rewarding. For instance, a study examining patient experience of a hospital renovation found that the changes to facilities led to enhanced patient experience, satisfaction and heightened quality of patient rooms and safety [17]. One study examining the opening of a new hospital in Canada found more favourable attitudes among staff towards the newly developed facility and an overall increase in workplace satisfaction and an improvement in workplace interactions immediately after the facility opened [18]. While there are many long-term benefits of revitalising hospital infrastructure, such as improved patient care and staff work environments [19], it is also the case that large-scale hospital change may be a stressful time for staff and ultimately disrupt the delivery of patient care. Although the literature has retrospectively identified the benefits of hospital redevelopment for both staff and patients, there is little research that seeks to understand their perspectives of the redevelopment (positive and negative), both before and after the redevelopment.

### Study aim

**Fig 1:**
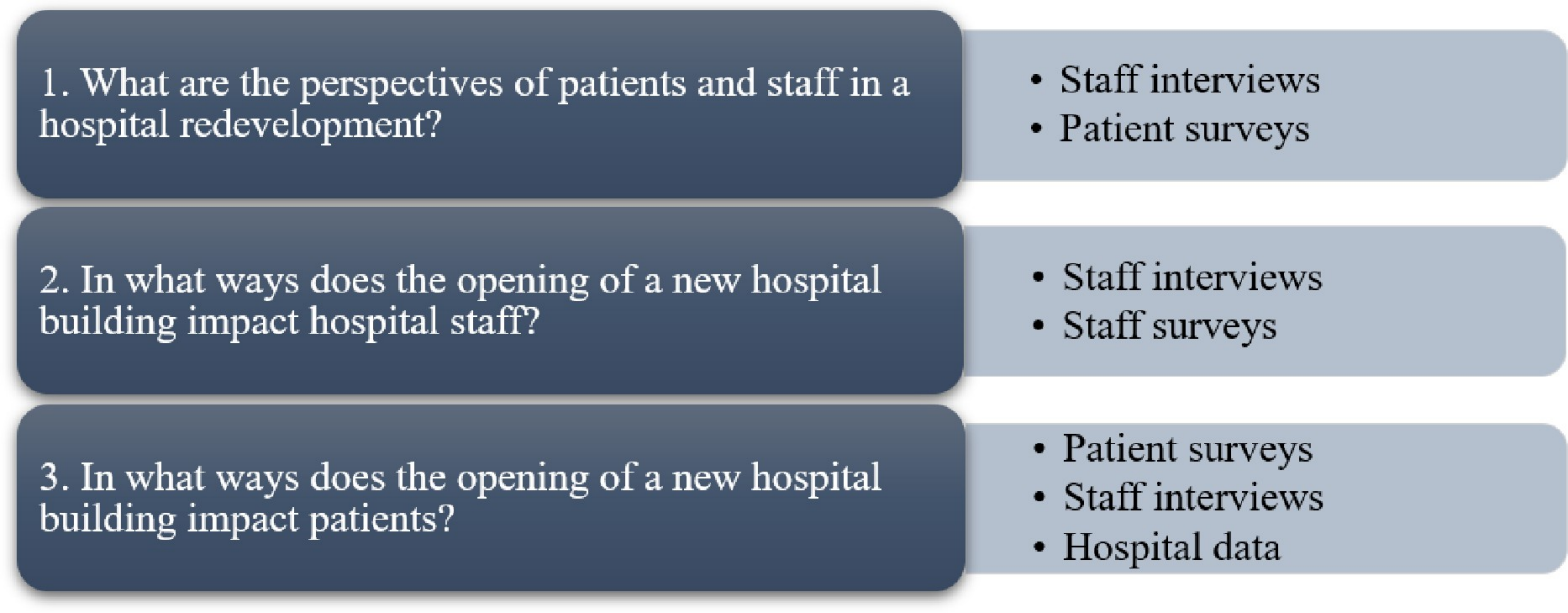
Research questions and study methods.

The aim of this study was to explore the perspectives and experiences of staff and patients before and after the opening of a new hospital building as part of a large public hospital redevelopment project in Sydney, Australia. Specific research questions are outlined in Fig 1. Based on previous research indicating benefits of redevelopment, we anticipated that the inconvenience of physically moving into the new building would quickly be replaced with positive sentiments around improved functionality and more pleasant surroundings [20, 21].

## Methods

The study employed a longitudinal mixed methods case study design consisting of a series of measurements over time: staff surveys, staff interviews, patient surveys, and hospital data. The case study approach was chosen given its value in analysing complex events with the capacity to obtain in-depth information about the topic at hand [22]. This study forms part of a larger program of research exploring how hospital redevelopment influences the wider context of the hospital and its functioning [23]. Ethical approval was granted by the relevant Ethics Committee in Australia (no: 18/233). Participation was voluntary and anonymous.

### Study site and participants

The research was conducted at a publicly funded hospital in metropolitan Sydney, Australia. At the time of data collection, the hospital was undergoing a multimillion-dollar development including the opening of a new hospital building and relocation of several units to this building (e.g., emergency department, maternity services, theatres, intensive care unit (ICU)). The development was multi-staged, with services opening at different points in time. More detail on the study setting are reported elsewhere [23, 24]. In this paper, we focus on data that were collected before (2019) and after a new hospital building was opened (2020), as part of a larger hospital redevelopment project that was ongoing.

**Fig 2:**
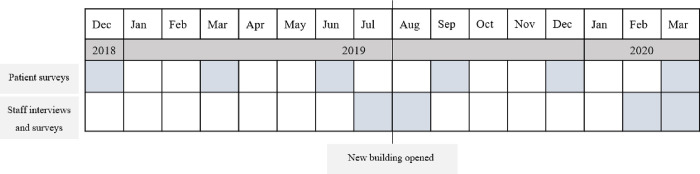
Timeline of data collection.

Participants were hospital staff (clinical and non-clinical) and patients. Staff surveys and semi-structured interviews were conducted before the building opened (July–August 2019) and then six months later, once some staff were working in the new facilities (February–March 2020; See Fig 2). Patient surveys were collected more frequently throughout the research project (T1-T6). Hospital data were collected as available (tri-monthly).

### Survey

A survey was distributed to staff at two time points, before and after the building opened. Surveys were distributed online and in paper-based form. Online surveys were distributed via email to participants from an online all-staff bulletin. Hospital staff were invited to participate by clicking on a link that led to the online survey powered by *Qualtrics* [25]. Similarly, paper-based surveys were distributed to hospital staff by ward managers and health professional directors.

The survey took 10–15 minutes to complete. Questions were extracted from validated questionnaires to capture perceptions of organisational culture [26], change readiness (appropriateness, change efficacy) [27], and staff-wellbeing, comprised of job satisfaction [28], burnout [29], and intention to leave [30]. The survey also included open-response questions designed to elicit additional information regarding staff experience of the organisational change. For example, “*Did the move into the new acute services building impact your work*? *If YES*, *why*?*”* The survey was piloted with an expert panel (n = 10; researchers with clinical backgrounds and hospital staff not involved as participants in the study) and modified where necessary to increase clarity.

### Interviews

Hospital staff were invited to participate in semi-structured interviews via a question on the survey as well as through purposive sampling by department heads and snowballing from participants. Interviews were conducted in private settings at the participants’ place of work (e.g., ward interview rooms, private offices) or over the phone. We used a semi-structured interview guide with questions that aimed to explore organisational culture, communication and collaboration, and the potential influence of the redevelopment on staff and patient experience. All interviews were conducted by CP, an experienced qualitative researcher with a background in psychology, and were audio-recorded and transcribed verbatim. The number of interview participants at each phase of data collection was determined by data saturation, which qualitative researchers consider usually occurs between 20 and 30 participants [31], but cannot be said as a rule to occur at any particular number.

### Patient experience survey

Patients at the hospital under investigation are routinely invited to participate in questionnaires sharing their experience of care via an SMS sent to their phone. For the series of questionnaires that spanned the duration of this research project, the patient survey was designed with additional questions pertaining to this study. This included routinely collected hospital experience questions at the hospital under investigation as well as a validated survey of patient experience: Picker Patient Experience Questionnaire-15 (PPE-15) [32]. The PPE-15 provides a global rating of the quality of healthcare based on in-hospital patient experiences. Higher scores (approaching 100) indicate a more positive patient experience. The survey also included open-response questions designed to elicit additional information regarding patient experience of the hospital redevelopment, for example: “*[name] Hospital is currently undergoing a large redevelopment project including the opening of a new acute services building in 2019*. *In what ways*, *if any*, *has this redevelopment affected your stay*?” The survey was distributed to cohorts of patients at six time points, three months apart, and took 5–10 minutes to complete.

### Supporting hospital data

Routinely collected hospital data of interest to this study were made available to the research team. Hospital data included: percentage of patients that started treatment on time, percentage of emergency department (ED) patients seen within clinically recommended time, and percentage of ED patients’ waiting times within four hours. Data were collected at tri-monthly intervals over two years.

### Analysis

First, each of the diverse data sets was analysed separately. For quantitative analysis, demographic and descriptive data (e.g., staff wellbeing, patient experience) collected from staff and patient surveys were analysed using SPSS version 25 [33]. Each of the surveys were post-weighted by age, sex and profession to reflect population distribution according to hospital records of employed staff at both stages of data collection. Weighting was conducted through a survey raking technique in XLSTAT [34]. Descriptive statistics were used to illustrate the characteristics of the samples as well as compare study variables over time using t-tests for continuous variables and chi-squared tests for categorical variables. Continuous variables were tested to confirm normality of data distribution prior to undertaking analysis. Due to the exploratory nature of the study and relatively small sample sizes, the significance level was set at p < .05 and not adjusted for multiple comparisons.

Qualitative data (open-responses from surveys of staff and patients and staff interview data) were analysed using thematic analysis [35] in NVivo version 12 [36]. Thematic analysis involved inductive coding to identify patterns emerging from the data. Through examination of codes and coded data, themes were developed. Coding was primarily conducted by the first author with frequent discussion of codes and themes with the broader research team throughout the analysis process. This process was conducted separately for the survey responses and patient survey responses in 2019 and 2020 to identify key themes of stakeholder experiences of a hospital redevelopment. For staff interviews, analysis of the first round of interviews is reported elsewhere [24]. The analysis of the second round of interviews included inductive coding together with deductive coding, based on the themes developed from the first round of data collection (before the opening of the new building). For the presentation of all qualitative data, extracts were edited minimally to enhance readability, without altering meaning or inference. Quotes from staff are provided with information on phase of data collection (2019 or 2020) and their profession (AD: Administrative staff; AHP: Allied health professional; DR: Medical staff; GS: General services staff; N: Nursing; MW: Midwifery staff; NUM: Nursing unit manager; OTH: Other profession; Ph: Pharmacy). Patients’ quotes are qualified with information on the time point of data collection (T1-T6, e.g., Patient1_T1).

Lastly, the different methods were integrated to form an overall picture of staff and patient experiences of a hospital related to the opening of a new building. We used triangulation techniques to synthesise the different methods and identify similarities or differences across datasets to ultimately aid in our interpretation of the findings.

## Results

### Descriptive statistics

The number of participants per data collection method are provided in Table 1. In regard to patient questionnaire data, most participants were female (*n* = 187; 75.7%) and over one third were aged between 18–34 years (*n* = 97; 39.3%) across all six time points. Staff interview participants were mostly nursing or midwifery professionals (2019 = 56.5%; 2020 = 40.0%). Other participants included medical staff, allied health professionals, general services personnel, hospital management, and administrative staff. On average, the first round of interviews were 17 minutes long (range: 7–33 minutes) and 18 minutes long for the second round (range: 8–27 minutes). For staff survey participants, see Appendix 1 for unweighted and weighted participant demographic and work characteristics. As shown (percentages in Appendix 1), post-weights were successful in taking into account differences in demographics and creating datasets that were appropriate for longitudinal statistical comparison.

**Table 1:** Participants per data collection method.

		Before opening of the new building– 2019 *N*	After building opened– 2020 *N*
Staff	Interviews	46	20
	Survey	153	139
Patient	Survey	122	133

### Themes and perspectives of staff throughout the hospital redevelopment

We identified four key themes and perspectives of staff with the opening of a new hospital building as part of a hospital redevelopment: (1) change uncertainty, (2) communication effectiveness, (3) staffing adequacy, and (4) staff resilience.

#### Change uncertainty

A major theme among participants’ responses throughout the hospital redevelopment was that it was a highly uncertain time for staff, both before the new building opened—“I just don’t know. I’m worried because I don’t know what we’re walking into” (N4_2019)—and after—“It was chaotic. I was in charge the first day we moved over–we had no idea where things were” (N2_2020). This uncertainty reported among staff was noticeable to patients: “Many nurses did not know where some items were due to the restructuring” (Patient1_T5). Factors contributing to staff feeling uncertain included: not feeling informed or involved in the hospital redevelopment; inconsistency in top management; and that the change was not static, but a dynamic, elongated transition: “Our go live plan was this one-week, Big Bang Theory, which ended up drawing out to four months of opening” (Ph1_2020).

#### Communication effectiveness

Communication effectiveness was identified as another significant theme by staff throughout the change. Where communication was perceived as successful, staff experiences were more positive and accepting of the move to the new building: “Staff were prepared for the relocation for more than a year” (N5_2020). Effective communication was reliant upon a champion of change, for example, a nursing unit manager that went to great efforts to ensure the staff had a positive attitude towards the move and knew what they were walking into: “I get the information, I pass on to all my team, and I communicate it as well in the staff meetings. Communication is the key, so I keep my staff updated” (NUM3_2020). This implies that where communication was effective, the move into the new building was smooth.

According to key stakeholders at the hospital, planning for the hospital redevelopment commenced five years prior to the opening of the new facilities and was managed by a project team. The process sought to involve staff by codesigning new models of care; soliciting their feedback on building design; hospital tours; and orientation to the new buildings and equipment.

Participation of staff in the early design phase was considered important: “Some areas could have been bit better if the clinicians were involved” (NUM2_2020); “Nurses had little/nil input in the design of the unit. Patients and staff would have had a better working environment and patient centred kind of building if input was requested” (N3_2020). For some, communication was more important than staffing levels: “We don’t need more staff. We need better communication… Just because you chuck in 200 nurses, that’s not going to be a solution, it’s just going to be more people added to the confusion of what’s already a confused mess” (N1_2020). One clinician commented:

*“I think that’s where a lot of it fell down*. *We didn’t necessarily have that structure*, *or it wasn’t known if we did have it*. *We didn’t know the pathways to escalate or to get responses*. *We didn’t necessarily know who was responsible for what or what they were supposed to deliver*” (Ph1_2020)

#### Staffing adequacy

Another key theme was staff perspectives regarding the adequacy of staffing to manage and cope with the change. This included concerns before the opening of the new building: “My biggest uncertainty at the moment is the fact that I’m really concerned about whether I’m actually going to get enough staff” (GS1_2019). Others considered that a “like for like” approach to directly transfer operations from one building to another without additional staff was inadequate to support optimal patient care:

“***They’ve got these fresh*, *new buildings that are poorly designed*, *and there’s not enough staff to manage the high acuity of patients coming through***.” (MW1_2020).

The notion of the development impacting patient care and experience after the building opened was flagged: “We are compromising clinical care because staff are overworked” (DR1_2020); and “You never know where anyone is, a lot of the time the desk is empty and there is no one to help you” (MW1_2020).

#### Staff resilience

Staff found ways to cope and make the change work. Staff interview responses recounted many experiences of adaptability and finding ways to cope with unexpected situations as they transitioned into a new place of work: “Staff are collaborating and working together to come up with ideas to sort through teething issues” (N2 _2020). Other staff found that although they had time to plan beforehand, it was inadequate: “We had deadlines that were changing and no additional resources to meet. What I would say were unachievable deadlines. So, we just have to do what was necessary to get there” (Ph1_2020).

### The impact of opening a new hospital building

#### Staff

**Fig 3:**
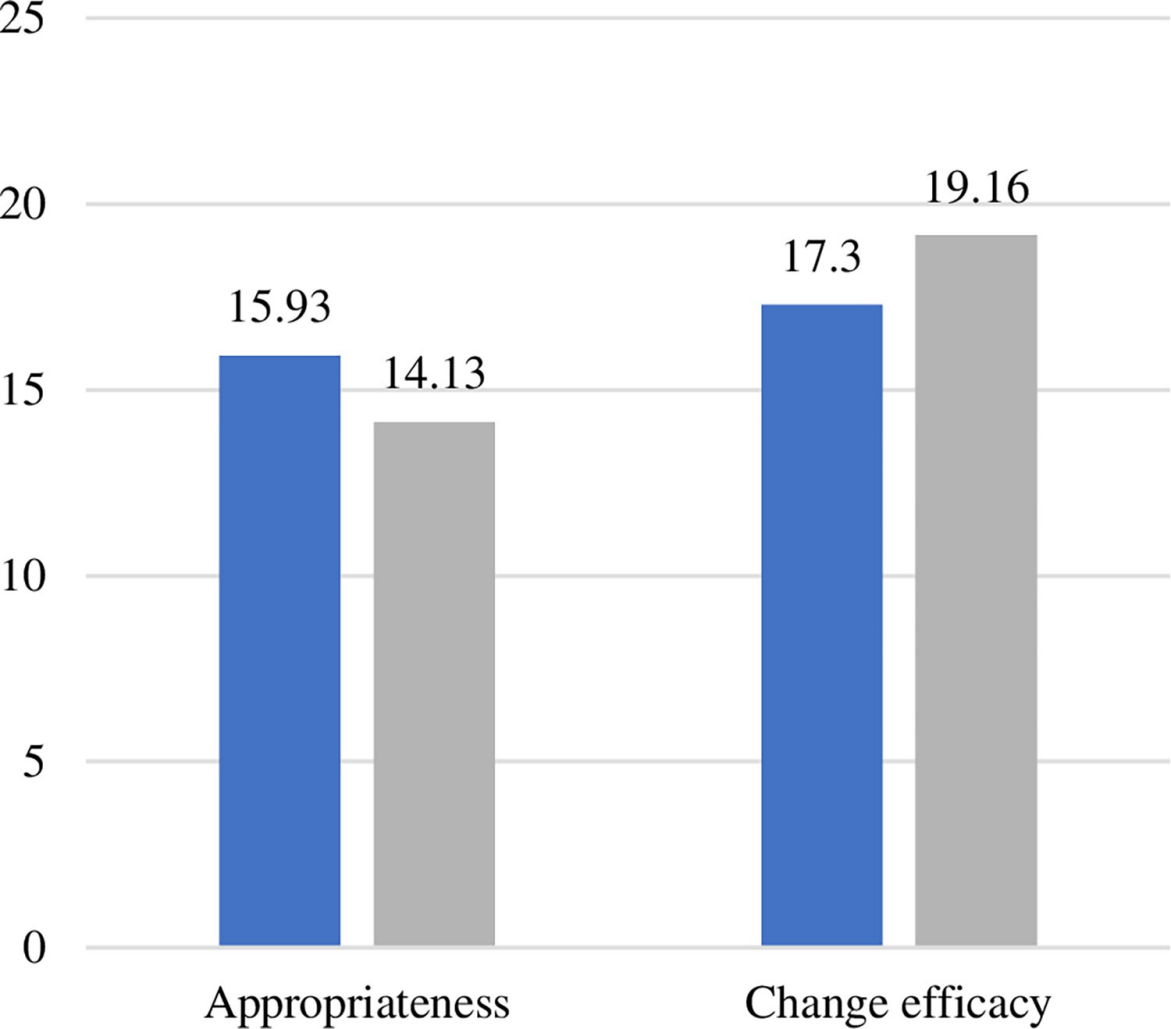
Significant difference in staff change readiness over time.

We found significant differences over time for perceptions of change readiness. Specifically, perceptions that the organisational change was appropriate significantly decreased (2019: 15.93 ± 3.86; 2020: 14.13 ± 3.62; *p* < .001) and perceptions that staff were capable of dealing with the change significantly increased (2019: 17.30 ± 4.77; 2020: 19.16 ± 4.36; *p* = .001) after the building opened compared to before (Fig 3).

We explored the differences in change readiness between staff that had over four years’ experience working at the hospital compared to those with less experience to investigate if staff who had been at the hospital during the design phase of the building (that took place five years prior) were more ready for the move. This finding was not significant, showing that despite experience at the hospital, and the potential to have been involved in the building design, there were no significant differences in staff experiences of change readiness.

**Fig 4:**
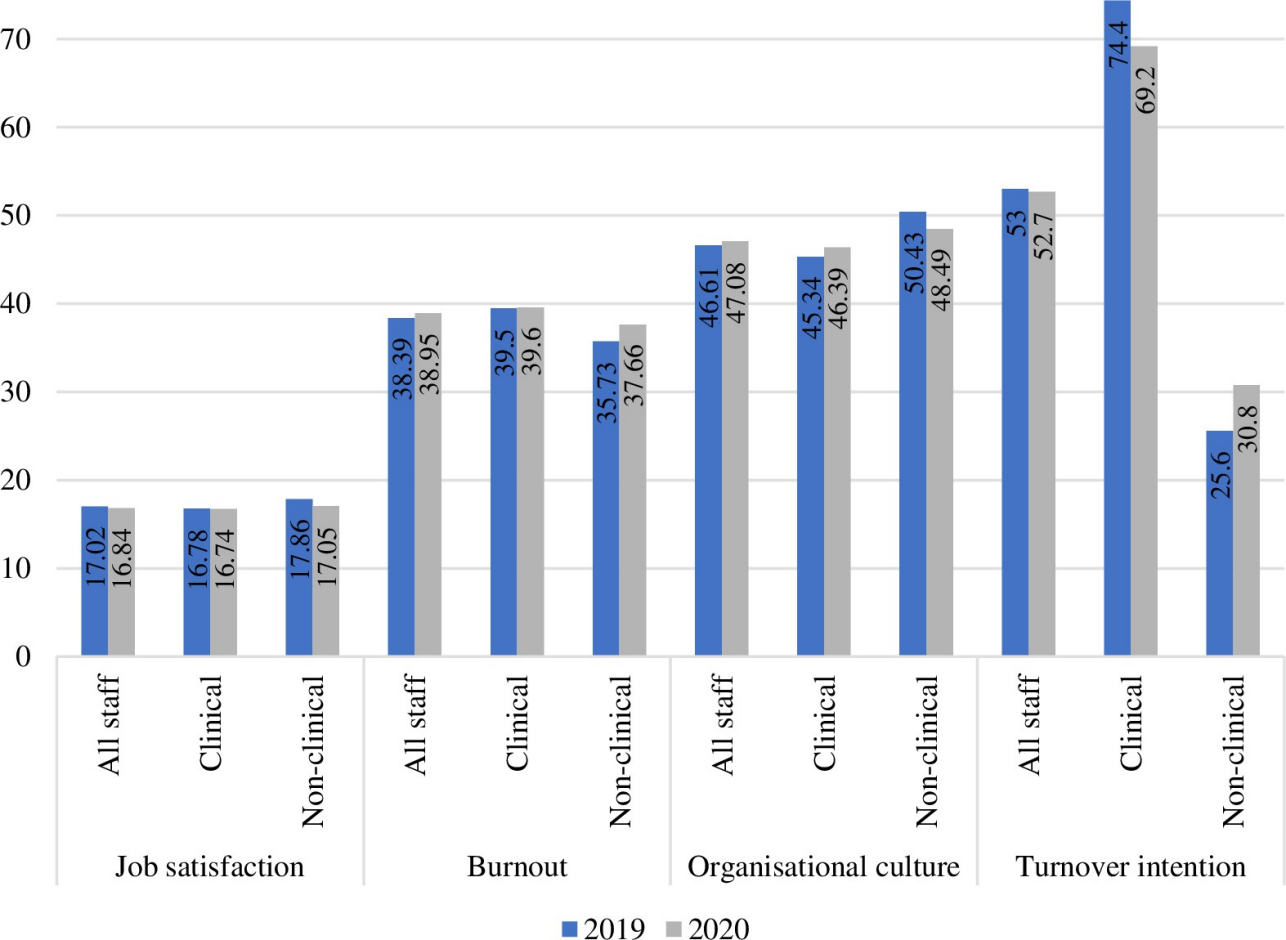
Staff outcomes over time. *Note*. * p < .05.

No statistically significant differences were found for staff burnout, job satisfaction, turnover intention, or organisational culture before, compared to after, the building opened. We looked specifically at differences in clinical versus non-clinical staff and found that job satisfaction was significantly lower for clinical staff compared to non-clinical staff in 2019 (Clinical: 16.78 ± 5.52; Non-clinical: 17.86 ± 4.58; *p* < .05), although the difference was not significant after the building opened (Clinical: 16.74 ± 5.64; Non-clinical: 17.05 ± 5.90; *p* = .401). Across both groups, there was a reduction in job satisfaction and an increase in burnout in 2020 compared to 2019 (although these findings were not significant). Further, there was a generally positive trend for perceptions of organisational culture, however, non-clinical staff perceived slightly more negative culture in the hospital after the building opened. Staff turnover intention was highest among clinical staff in 2019, with 74.4% indicating they intended to leave in the coming year (similar to the 69.2% in 2020). All differences in staff outcomes in 2019 and 2020, with comparisons between clinical and non-clinical staff, are displayed in Fig 4.

There were several potential reasons identified in the interview data for why some staff felt unaffected throughout the hospital redevelopment. First, not all staff were working in the new building. These staff were, for the most part, unaffected and unaware of the others feeling unsettled: “It has not really made a difference in terms of my workflow. I think that’s because my work didn’t extend physically into the [new] building” (AHP1_2020). Some staff working in the new building felt similarly unaffected by the transition because they were adequately informed and prepared for the change: “I prepared all my nursing staff prior to the move, and that has assisted us in a smooth transition” (NUM 3 _2020).

Although we found no statistically significant differences in staff burnout, job satisfaction, or turnover intention before compared to after the new building opened, analysis of interview data revealed that for some staff, the period after the building opened was a particularly challenging time: “Morale has been terrible, absolutely terrible” (N6_2020); “You become a nurse because you want to help people … highly destructive to both the medical and the nursing staff, if they haven’t got the tools to be able to care for our community” (N1_2020). Staff reported immense pressure in having to adapt to a new space that they felt inadequately prepared for: “Staff struggled a lot. There wasn’t enough orientation time, and they were thrown into the ward with maybe one tour of the unit and then a week later, they were working on the ward. So that was really stressful” (NUM4_2020). A possible contributor to experiences of stress and burnout among staff was navigating the physical changes: “I think people are finding it quite challenging getting their head around the changes to workflow, the change to positioning, the changes of just the location, the difference in distance” (OTH1_2020).

#### Patients

Overall, patients reported that they were always treated with kindness and respect (*n* = 176; 69.0%) and were extremely likely to recommend the hospital to family or friends (*n* = 155; 60.8%). These positive sentiments (both quantitative and qualitative)—e.g., “The maternity ward being one bedroom was a dream” (Patient1_T4)—did not differ over time and were consistent with staff reflections: “For patients, it’s a better quality of care, better service that we are providing here” (NUM3_2020).

Patients had both positive and negative perspectives on the redevelopment: “These big redevelopment projects have no value if you can’t provide satisfied service to a patient” (Patient2_T6). Patients were generally positive toward the capital investment of the building: “It was a new building, and the facilities were excellent! It’s like I was admitted in a hotel” (Patient1_T4); “The facility was great everything looked good” (Patient2_T5).

Some patients described a poor experience from the construction work which potentially acted as barrier to having a restful and healing experience: “The noise from construction made it difficult to rest and recover from surgery” (Patient3_T5).

Throughout the redevelopment, patients attributed some negative aspects of their care experience to perceptions of staff stress: “Staff seemed stressed and under resourced which manifested in unsatisfactory care which was the worst aspect of the inpatient experience” (Patient1_T6); “Seemed a bit understaffed—it took me seven hours to get some information I needed due to there being an overpopulation and not enough nurses” (Patient1_T5); “Radiology staff seemed stressed and under resourced which manifested in unsatisfactory care which was the worst aspect of the inpatient experience” (Patient1_T6); “The communication between staff is horrendous, midwives, admin staff and doctors need to communicate more about their patients” (Patient1_T4).

According to key stakeholders at the hospital, the opening of the new building resulted in longer transit times for patients between wards and treatment areas, and increased delays in accessing porters. This finding was consistent with other forms of data collected in this study. Firstly, hospital data showed that ED patients experienced longer waiting times to be seen within a clinically recommended time, immediately before and once the new building opened before stabilising within a few months. The percentage of patients commencing treatment on time, initially dropped before rebounding to the pre-move levels within a few months of opening. Patients reported experiences of increased wait times in the emergency department, as did staff: “Patient satisfaction is bad, the anger in the waiting room is palpable” (N1_2020).

**Fig 5:**
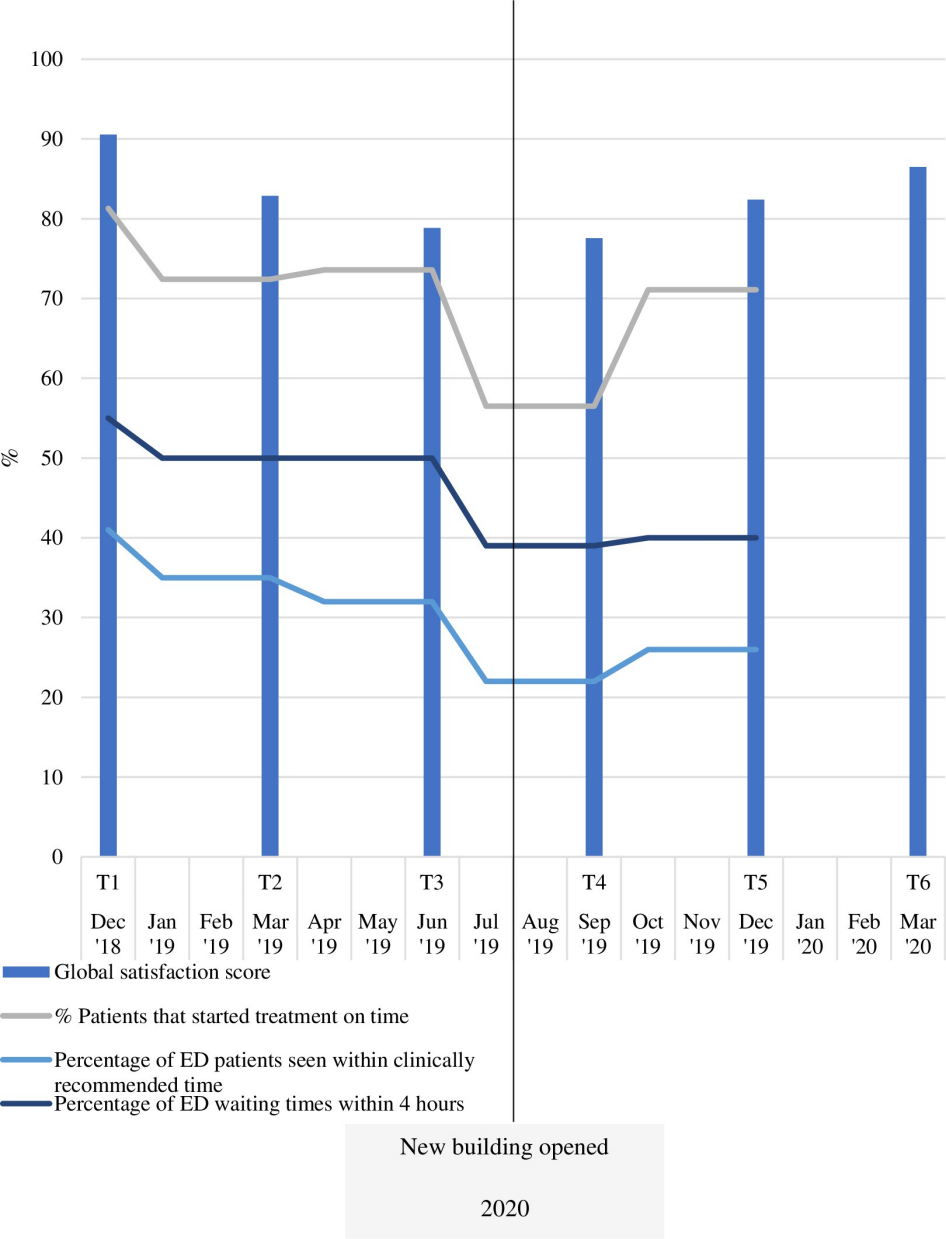
Patient experience over time.

Fig 5 shows that global satisfaction scores from patient survey data showed that patient experience significantly declined after the building opened compared to before (2020: 81.70 ± 21.52; 2019: 84.43 ± 18.46)), *t*(254) = -64.55, *p* < 0.05. Fig 5 also flags that global satisfaction scores improved a few months after opening of the new facilities.

## Discussion

The present study aimed to explore the perspectives and experiences of staff and patients before and after the opening of a new hospital building as part of a large hospital redevelopment project. The findings shed light on the challenges for staff working in a hospital undergoing change and how the move into a new building can disrupt patient care. We identified four key themes and perspectives: change uncertainty, communication effectiveness, staffing adequacy, and staff resilience; and revealed that the move into the new hospital building was challenging for staff and, although positively received by patients, did negatively impact patient experience.

We showed that while initially staff felt excited for the opening of the new hospital building, this was shortly overridden with a sense of disillusionment for some once they moved in. This is consistent with past reports of staff experience working in a new hospital building [37]. The sense of disappointment reported by staff was reflected in the finding that with the move into the building, staff perceptions of the appropriateness of the change significantly decreased (i.e., that the change was legitimate, that the organisation would benefit from the change, and that the change would improve the organisation’s overall efficiency). This was consistent with frustrations expressed by staff regarding the challenge to working efficiently in a new building. Coupled with poorer job satisfaction and high levels of turnover intention both before and after the opening of the new building, these findings point to a stressed workforce.

Staff perceptions as to the adequacy of staffing during the period of change was highlighted in this study. Factors contributing to these perceptions may include the numbers and skill-mix of staff available to provide care; the impact of burn-out and job dissatisfaction on the provision of care; professional considerations as to what constitutes quality of care; and the organisation of care delivery. Further research into workload and resource planning tools to investigate staffing adequacy is required.

Notably, staff perceptions of change efficacy significantly increased (i.e., not anticipating any problems adjusting, having the skills needed to make the change work), indicating that staff found ways to cope and make the change work, despite their initial doubts before the building opened. This highlights the resilience of staff as they overcame challenges and found ways to deliver patient care in the new facilities even with the many challenges they faced (e.g., understaffing, equipment not being in the right places). This finding is consistent with the resilient healthcare literature which discusses the common experience among healthcare staff to ‘make do’ in unsuitable hospital workspaces, and find ways to circumvent or overcome challenges that arise through, for example, working together and improvising [38, 39]. Research from our colleagues and collaborators suggests that resilience training is one way to ensure staff have appropriate expectations (i.e., anticipating the disruption that will come with the opening of a new building) and are prepared from the outset [40]. Project managers should consider investing in resilience training as part of the preparation for hospital redevelopment to enhance productivity and flexibility throughout the change.

The research identified the challenge of communicating about the change when many factors were uncertain. This was specifically the case given that timeframes for opening the building and staffing models were different to what was originally planned and thus anticipated by staff. Staff expected all facilities to be opened concurrently rather than in a planned sequential staged approach. Conversely, positive staff experiences of working in the building were often linked to open and transparent communication from department managers. For example, some nursing unit managers coordinated frequent face-to-face meetings to update staff, even if that meant reiterating that certain aspects of the change were unknown. This is consistent with organisational change literature that highlights the importance of open communication across all levels of staff during hospital organisational change [41].

Further, patient global satisfaction scores significantly declined after the building opened compared to before and improved a few months after opening. There are many potential demand and supply factors that may have contributed to this result, over and above the move to the new building. Increased demands on the hospital include factors such as the timing of the moves in mid-winter associated with seasonal increases in the numbers of patients and staff influenza, as well as the initial attractiveness of new facilities as a destination for patients to receive care.

In service provision, there are multiple possible explanations to account for poorer patient experiences during the move to the new building. One explanation of this is that during the complex and busy time of moving into the new building, staff were distracted, focused on orientating themselves in the new environment and making things work. Specific design issues or a lack of equipment where needed may have made delivering patient focused care a challenge. More attention to how clinicians work on the frontline during the design phase may help make the transition less challenging for staff (e.g., ensuring wards and departments are stocked from day one with the equipment needed for day-to-day work). The transition time from designing to building commissioning and opening was a lengthy process, with design occurring five years prior to the building opening. Given that over 40% of the staff who participated in the survey were employed at the hospital for less than four years, this study highlights the importance of continuous staff input throughout this lengthy process. This is consistent with past research that found that consulting staff increased the effectiveness and efficiency of the newly developed areas as staff input helped ensure that the new spaces met the needs of both staff and patients [42].

### Implications

Learning from this study, we present key recommendations for how future hospital redevelopment projects could be improved to enhance support for staff and patients as they move into new hospital buildings (See Box 1). These key recommendations are targeted towards policy makers, hospital managers and hospital staff.

**Box 1. Lessons learned for future hospital redevelopment projects**

- Integrate design with how people work—include more frontline clinicians throughout the entire process of design and building new hospitals.
- Ensure building designs are cognisant of operationally efficient staff ratios for the patient care environment.
- Base financial operational decisions to support new infrastructure on realistic activity projections; conservative efficiency savings; and acknowledgement of the impact of increased floor space and travel distance time between units on cleaning and transportation for staff and patients.
- Consider additional investment in quality improvement, research and innovation in the workplace.
- Fix dates for the staged operational commissioning and opening of specific units with sufficient lead time that does not change; with clear communication of these dates to all staff.
- Recognise that new models of care mean new ways of staff working within new facilities and equipment. New ways for staff working should be recognised as more significant than familiarisation with a new environment, with staff trained in new practices required prior to opening of new facilities.
- Invest in staff resilience training early.
- Identify and support champions of change to keep staff informed and morale high throughout the change process.
- Inform consumers and stakeholders as to what is being done to reduce the impact on the care provided and patient experience with the opening of new facilities. Expectations need to be set that further redesign of work practices will be required after opening new facilities to optimise patient care and experience.

### Strengths and limitations

This is the first Australian case study of hospital redevelopment, and one of the few internationally to examine the perspectives, experiences and impact of the opening of new hospital facilities on staff and patients. A notable strength is the use of mixed methods to capture the breadth of experiences of staff and patients. However, a limitation of this study is that findings may be restricted to the contextual subtleties of the hospital and the specificities of the hospital redevelopment. Further, due to the exploratory nature of the results and the relatively small sample sizes, the significance level of .05 was not adjusted for multiple comparisons. This means that we cannot make strong conclusions on the observed statistically significant differences, and the results need to be interpreted with caution until they have been replicated in follow-up research. Finally, given that the survey was advertised via email within the hospital bulletin and paper-based surveys were distributed to hospital staff by ward managers and health professional directors, we were unable to calculate a response rate. Nevertheless, the study was designed to produce nuanced, in-depth data with aspects transferable to other instances of large-scale hospital change. The research is applicable to other hospitals, particularly in Australia’s most populous state, New South Wales, where there are approximately 30 large public hospitals that have similar organisational structure in terms of funding, administration and staff skill mix. Jurisdictions in Australia and other countries are undergoing similar building programs [6, 7, 19].

Further, another strength of this study is that we collected data from a variety of staff (clinical and non-clinical) from different departments and wards in the hospital. However, with that comes the limitation that contextual nuances of how different staff in different departments or wards experienced the change have been amalgamated and reported as a generic experience. To mitigate this, in the write-up of study findings we have highlighted where differences were observed (e.g., some staff felt more informed about the change from their nursing unit managers compared to others).

## Conclusion

Moving into a new hospital building can be particularly challenging for staff and patients. For many this time was characterised by uncertainty, where staff displayed practices of resilience to deliver patient care during a challenging period of change. This paper highlights the complexity of organisational change in hospitals and the importance of supporting staff and patients during the opening of a new hospital building as part of a large hospital redevelopment project.

## Abbreviations

AHP: Allied health professional
DR: Medical staff
ED: Emergency Department
GS: General services staff
ICU: Intensive Care Unit
MW: Midwifery staff
N: Nursing staff
NSW: New South Wales
NUM: Nursing unit manager
OTH: Other profession
Ph: Pharmacy
PPE-15: Picker Patient Experience Questionnaire-15
